# Editorial: CNS immunity – paradox of maintenance, repair and destruction

**DOI:** 10.3389/fimmu.2026.1954884

**Published:** 2026-08-18

**Authors:** Anirban Ghosh, Roman Sankowski, Sagar Acharya, Karen Bohmwald

**Affiliations:** 1 Cell Development and Immunobiology Laboratory, Department of Zoology, School of Sciences, Netaji Subhas Open University, Kolkata, West Bengal, India; 2 Institute of Neuropathology, Faculty of Medicine, University of Freiburg, Freiburg, Germany; 3 Cellular and Molecular Immunology Laboratory, Department of Zoology, Vidyasagar University, Midnapore, West Bengal, India; 4 Institute of Biomedical Sciences, School of Health Sciences, Universidad Autónoma de Chile, Santiago, Chile

**Keywords:** advanced neuroimaging techniques, blood brain barrier (BBB), gut-brain axes, neuroimmune-endocrine-microbiota network, neuroimmunology and microglia, transcriptomics

The trajectory of neuroimmunological research over the past century exemplifies the paradox of repair, maintenance, and destruction that defines CNS immunity. The discovery of microglia by Pío del Río-Hortega in the 1920s marked the first recognition of resident immune cells within the brain, challenging the prevailing notion of the CNS as an entirely immune-privileged site (1). For decades, microglia remained largely overlooked, until the renaissance of interest in the 1970s–1980s led by Krutzberg, Lassmann, and Perry, who employed histological and ultrastructural methods to demonstrate microglial activation in neurodegenerative conditions and traumatic brain injury (2, 3). These early studies, relying on microscopy, immunohistochemistry, and Western blotting, revealed microglia as central players in Alzheimer’s disease, brain aging, and other CNS disorders, laying the foundation for modern neuroimmunology. By the late 1990s, other groups including Kettenmann and colleagues expanded this paradigm, showing microglial involvement in gliomas and brain tumors, reframing them not only as custodians of homeostasis but also as accomplices in pathology (4).

The conceptual leap from descriptive pathology to functional immunology was catalyzed by the identification of microglial polarization states—M1 (pro-inflammatory) and M2 (anti-inflammatory)—through flow cytometry, transgenic mouse models, and transcriptomic profiling (5). Although later recognized as oversimplified, this binary classification provided a framework to dissect microglial heterogeneity. Advances in RNA sequencing, particularly single-cell RNA-seq, revealed a spectrum of microglial phenotypes beyond the M1/M2 paradigm, highlighting context-dependent transcriptional programs in disease and development (6–8). Parallel innovations in two-photon microscopy and live-cell imaging enabled dynamic visualization of microglial surveillance, synaptic pruning, and interactions with neurons and astrocytes *in vivo* (9). These methodological revolutions transformed microglia from static histological curiosities into dynamic, multifunctional regulators of CNS immunity, bridging cellular biology with systems-level insights.

Concurrently, the dogma of CNS immune privilege underwent profound revision. The rediscovery of the glymphatic system and meningeal lymphatics demonstrated active communication between the brain and peripheral immune compartments (10, 11). The blood-brain barrier (BBB) and cerebrospinal fluid (CSF) interfaces, once viewed as impermeable, are now recognized as selective gateways for antigen exchange, immune cell trafficking, and bidirectional signaling. Pathways via the olfactory and vagus nerves, coupled with neuroendocrine axes, further integrate CNS immunity with systemic physiology. The gut–brain axis, mediated by microbiota-derived metabolites and immune signaling, exemplifies this systemic interconnection, linking peripheral inflammation to neurodegeneration and psychiatric disorders (12). Thus, CNS immunity is no longer confined to the parenchyma but embedded within a broader neuro-immune-endocrine-microbiota network, where repair and destruction coexist in delicate balance.

Modern neuroimmunology now thrives at the intersection of molecular biology, neuroimaging, and computational systems science. High-throughput platforms—single-cell and spatial RNA-seq, proteomics, and metabolomics—map cellular diversity with unprecedented resolution, while advanced neuroimaging modalities (CT, MRI, and PET) integrate structural and functional data. Machine learning and AI-driven analytics synthesize these datasets, enabling predictive modeling of disease trajectories and therapeutic responses (13). Systems biology approaches reveal evolutionary interrelations of CNS immunity, situating human brain disorders within a continuum of adaptive and maladaptive immune responses. This transformation—from microglial discovery to systemic integration—revealed the paradox at the heart of CNS immunity: mechanisms that maintain homeostasis and enable repair can, under chronic dysregulation, drive destruction. On the one hand, icroglia were identified as protective in the case of Amyloid-beta phagocytosing disease-associated microglia (MGnD or DAM) (14, 15). On the other hand, as maladaptive tumor-associated macrophages microglia are involved in glioblastoma-associated immune suppression and mortality (16). These studies exemplify how the niche determines divergent functional immune states.

The present volume exemplifies the breadth of modern neuroimmunology, moving from cellular and molecular mechanisms to systemic integration. It captures the paradoxical nature of CNS immunity—where the same processes that maintain homeostasis and enable repair can, under chronic dysregulation, drive destruction. Cross-disciplinary perspectives linking neuroimmunity with vascular biology, tumor immunology, microbiota, and neuroendocrine signaling in this collection of papers demonstrate that CNS immunity is no longer isolation but as part of a dynamic neuroimmune-endocrine-microbiota network.

Cavalcanti et al., in their review “Neuroinflammation: targeting microglia for neuroprotection and repair after spinal cord injury,” dissect the paradoxical role of microglia in SCI, where they showed how early activation clears debris and supports axonal growth, but chronic signaling unleashes TNF-α, IL-1β, and IL-6, driving secondary injury and BBB breakdown. Using information gathered from the literature, they reveal microglial phenotypes as a dynamic continuum rather than binary M1/M2 states. The review highlights translational strategies—small molecules, biologics, exosomes, and BBB stabilizers—to reprogram microglia toward repair. The punchline is that microglia are both protectors and perpetrators, and precision modulation is key to meaningful recovery.

Preeclampsia is a multisystem hypertensive disorder of pregnancy in which defective trophoblast invasion and placental ischemia initiate a cascade of anti-angiogenic and pro-inflammatory signals that extend beyond the maternal-fetal interface. Interestingly, Liu et al. redefine preeclampsia as a neuro-immune-vascular disorder in their review “The neuro-immune axis in preeclampsia: from the maternal-fetal interface to systemic dysregulation,” moving beyond placental angiogenic imbalance to highlight dysfunctional crosstalk between neural circuits, immune cells, and vascular tone. They show how sympathetic overactivity, vagal dysregulation, and neuropeptide signaling amplify placental hypoxia, endothelial injury, and systemic inflammation. Using single-cell and spatial omics, the review uncovers aberrant dNK, macrophage, and Treg phenotypes at the maternal–fetal interface, as well as neuro-immune modulation in the bone marrow and spleen. Critically, CNS inflammation and BBB disruption emerge as drivers of seizures and long-term cerebrovascular risk. They conveyed the message that PE is sustained by feed-forward neuro-immune loops, demanding biomarker-guided neuro-modulatory therapies. Anirban Ghosh edits both of these papers.

The global crisis of antimicrobial resistance highlights the critical need for host-directed therapies that augment, rather than supplant, conventional antibiotics (17, 18). Present study by Seele et al. in the volume edited by Sagar Acharya establishes that glatiramer acetate (GA)—a synthetic polypeptide safely utilized for over twenty years to treat relapsing-remitting multiple sclerosis (MS) (19, 20)—acts as a potent enhancer of innate immunity. The investigators demonstrate that GA significantly accelerates the phagocytosis and intracellular killing of encapsulated *Escherichia coli* K1 by primary macrophages and microglial cells.

Mechanistically, this immunostimulatory effect depends fundamentally on the interleukin-10 (IL-10) pathway. While IL-10 is traditionally viewed as an anti-inflammatory suppressor of immunity (21), this study challenges that dogma. Antibody-mediated neutralization of IL-10 partially blocked GA-induced microglial phagocytosis, while ex vivo evaluations confirmed that *in vivo* GA pre-treatment successfully primed macrophages from wild-type mice but completely failed in strains. This underscores the highly complex regulatory role of cytokine networks in host defense (21, 22). Authors shows that GA successfully restores defective phagocytic capacity in macrophages from aged animals, addressing the challenges of immunosenescence (23) and drives bacterial clearance without triggering a toxic release of pro-inflammatory cytokines like TNFα. Anticipated future proof-of-concept studies in animal infection models remain essential to establish GA as a viable clinical countermeasure to limit antibiotic consumption.

Recent advances in neuroimmunology increasingly rely on precision medicine approaches that integrate molecular biomarkers, computational biology, multimodal neuroimaging, and targeted immunotherapies to improve diagnosis, prognosis, and therapeutic decision-making. Immunological profiling has already identified biological signatures that may facilitate earlier diagnosis, distinguish disease subtypes, and support treatment selection in multiple sclerosis (MS) (24). In parallel, contemporary research has emphasized that interactions among peripheral inflammation, glial responses, neuronal dysfunction, and neurodegeneration must be considered when developing individualized strategies for MS management (25).

Pan et al., in their original research entitled *“Machine learning and single-cell RNA sequencing analyses identify MS-related monocytes and a five-gene candidate biomarker signature,”* integrated single-cell RNA sequencing, high-dimensional weighted gene co-expression network analysis, and seven machine-learning algorithms to characterize disease-associated monocyte populations in experimental autoimmune encephalomyelitis. Their analyses identified COX5A, CTSS, GBP2, IRF7, and PGAM1 as a five-gene candidate biomarker signature associated with MS-related monocytes. The study further combined cell–cell communication analysis, transcriptional regulatory network inference, immune infiltration analysis, and experimental validation to investigate the biological relevance of these candidate genes. This integrative strategy is consistent with the emerging precision-neuroimmunology framework, in which immune signatures are used to improve disease classification and potentially guide therapeutic decisions (24). The focus on monocyte-associated molecular programs is also biologically relevant, as innate immune activation contributes to inflammatory tissue injury and interacts with neuronal and glial mechanisms implicated in MS-associated neurodegeneration (25). Nevertheless, the proposed signature should be regarded as a candidate biomarker panel requiring further validation in larger, prospectively recruited human cohorts before clinical application.

Complementing this molecular approach, Yang et al. in *“EEG biomarkers of microstructural damage in normal-appearing white matter among patients with neuromyelitis optica spectrum disorder: A DTI-EEG combined study* integrated diffusion tensor imaging and resting-state electroencephalography to investigate whether electrophysiological measures could reflect white matter microstructural damage in neuromyelitis optica spectrum disorder (NMOSD). The authors identified abnormalities in 17 of the 20 principal white matter tracts evaluated and found that several EEG functional connectivity indices, particularly coherence and phase-locking value measures in the theta and gamma frequency bands, correlated with fractional anisotropy. These electrophysiological indices were also associated with the number of attacks, disability, serum glial fibrillary acidic protein concentrations, and cognitive performance. The study therefore suggests that resting-state EEG may provide a comparatively accessible complementary measure of structural damage, although it cannot yet replace diffusion imaging and requires independent validation. This multimodal approach aligns with current efforts to integrate imaging, fluid, and functional biomarkers to improve diagnosis, relapse surveillance, disability prediction, and treatment monitoring in NMOSD (26). Recent reviews have similarly emphasized that contemporary NMOSD research is moving toward integrated disease characterization based on immunological, imaging, and clinical features rather than reliance on a single biomarker or outcome measure (27).

The translation of precision neuroimmunology into therapeutic decision-making is further illustrated by Liu et al., who compared eculizumab and rituximab in patients with aquaporin-4 immunoglobulin G-positive NMOSD. The study evaluated not only conventional disease-related outcomes but also visual acuity, visual-field impairment, motor function, spasticity, anxiety, depression, and social functioning over six months. Both treatment groups improved during follow-up, but patients receiving eculizumab showed greater improvement across several visual, motor, psychological, and social outcomes. These findings are mechanistically relevant because eculizumab directly inhibits complement component C5, whereas rituximab acts through B-cell depletion. Complement activation is a central effector mechanism in aquaporin-4 antibody-positive NMOSD, and complement-targeted monoclonal antibodies are among the principal mechanism-based therapies developed for this disease (27). However, because the study was retrospective and treatment allocation was not randomized, the reported superiority of eculizumab should be interpreted cautiously and confirmed through prospective comparative research. Even so, the inclusion of visual, motor, psychological, and social outcomes is valuable because relapse frequency alone does not fully capture the functional burden experienced by patients with NMOSD.

Collectively, these three studies illustrate complementary dimensions of contemporary precision neuroimmunology. Pan et al. address molecular stratification using single-cell transcriptomics and machine learning; Yang et al. examine multimodal monitoring via electrophysiology and diffusion imaging; and Liu et al. evaluate mechanism-based treatment using patient-centered functional outcomes. Together, they show that meaningful progress in autoimmune CNS disorders requires integrating molecular, structural, functional, and clinical information. Current precision-medicine frameworks similarly emphasize that biomarkers should contribute not only to diagnosis but also to disease-subtype identification, prognosis, treatment selection, and longitudinal monitoring (24, 26). The future challenge will be to validate these approaches in larger and more diverse cohorts, standardize analytical pipelines, and determine whether multimodal biomarker combinations improve outcomes beyond established clinical measures.

Liu et al., “Immune imbalance in the human hippocampus in PTSD revealed by single-nucleus transcriptomics”, focused on the role of immune imbalance in post-traumatic stress disorder (PTSD). The role of the brain immune system is increasingly recognized in this generally under-diagnosed condition. Using single-nucleus RNA-Seq on postmortem tissues, the authors identified reactive cell states across astrocytes, microglia, endothelial cells, and mural cells. In-depth trajectory and cell-cell interaction analyses showed maladaptive cell responses in astrocytes and microglia. These findings underscore the role of the immune system in PTSD and warrant further studies for successful clinical translation. Roman Sankowski edits this paper.

These seven papers collectively demonstrate that CNS immunity is a multifaceted paradox: mechanisms that maintain homeostasis and enable repair can, under chronic or maladaptive conditions, drive destruction. The volume highlights how modern neuroimmunology has evolved from descriptive pathology to integrative systems biology, incorporating omics, imaging, AI, and microbiota research. The future lies in precision interventions that balance repair with prevention of immune-mediated damage, guided by biomarkers, computational modeling, and evolutionary insights. This Research Topic provides a comprehensive roadmap for CNS immunity research, bridging cellular mechanisms, systemic physiology, and clinical translation, and embodying the paradox at the heart of CNS immunity.

Future progress will depend on embracing this paradox, leveraging integrative technologies to disentangle the duality of CNS immune responses, and translating insights into precision therapies. Biomarker-guided patient stratification, neuro-modulatory interventions, and AI-enabled predictive models will be central to this effort. The challenge is to balance the protective and regenerative potential of CNS immunity with its destructive capacity, ensuring that therapies harness the former without unleashing the latter.
