# Correction: Editorial: CNS immunity – paradox of maintenance, repair and destruction

**DOI:** 10.3389/fimmu.2026.1972861

**Published:** 2026-09-16

**Authors:** Anirban Ghosh, Roman Sankowski, Sagar Acharya, Karen Bohmwald

**Affiliations:** 1 Cell Development and Immunobiology Laboratory, Department of Zoology, School of Sciences, Netaji Subhas Open University, Kolkata, West Bengal, India; 2 Institute of Neuropathology, Faculty of Medicine, University of Freiburg, Freiburg, Germany; 3 Cellular and Molecular Immunology Laboratory, Department of Zoology, Vidyasagar University, Midnapore, West Bengal, India; 4 Institute of Biomedical Sciences, School of Health Sciences, Universidad Autónoma de Chile, Santiago, Chile

**Keywords:** advanced neuroimaging techniques, blood brain barrier (BBB), gut-brain axes, neuroimmune-endocrine-microbiota network, neuroimmunology and microglia, transcriptomics

In the published article, there was an error in the affiliation for author “Sagar Acharya”. As published, the affiliation was listed as “Instituto de Ciencias Biomédicas, Facultad de Ciencias de la Salud, Santiago, Chile.” The correct affiliation reads “Cellular and Molecular Immunology Laboratory, Department of Zoology, Vidyasagar University, Midnapore, West Bengal, India.”

The original version of this article has been updated.

